# Self‐reported sleep health characteristics may capture attention, memory, and global cognitive performance in older African‐Americans

**DOI:** 10.1002/alz.095038

**Published:** 2025-01-09

**Authors:** Stanley Romain, Payton G. White, Bernadette A. Fausto, Mark A. Gluck

**Affiliations:** ^1^ Rutgers University–Newark, Newark, NJ USA

## Abstract

**Background:**

Older African Americans bear a disproportionate burden of both sleep problems and cognitive dysfunction. Alarmingly, poorer sleep health may put an individual at higher risk of cognitive dysfunction. However, the relationship between sleep and cognition in older African Americans continues to be understudied. This study aimed to examine the relationship between subjective sleep health characteristics and cognitive function among older African Americans.

**Method:**

62 older African American participants (mean age = 72.19; SD = 6.651; mean education = 14.28; SD = 6.651; 51 female; 11 male) from the *Pathways to Healthy Aging in African Americans* study completed a demographic questionnaire as well as cognitive assessments of attention, episodic memory, executive function, language, and global cognition. The following sleep health characteristics were derived from the Pittsburgh Sleep Quality Index: Subjective sleep quality; sleep latency; sleep duration; sleep disturbances; use of sleeping medications; daytime dysfunction; and global index score. Hierarchical linear regression analyses were employed to investigate the relationship between sleep health characteristics and cognitive function while controlling for age, sex, education, waist‐to‐hip ratio, and depressive symptomology.

**Result:**

The relationship between subjective sleep quality and episodic memory (*B* = ‐1.715; *p* < 0.001); sleep disturbances and global cognition (*B* = ‐1.581; *p* = 0.020); sleep latency and global cognition (*B* = ‐1.033; *p* = .011); and sleep efficiency and attention (*B* = 0.249; *p* = 0.038) were significant before and after controlling for covariates. Overall, poorer sleep health across multiple characteristics was associated with worse cognitive function.

**Conclusion:**

Self‐reported sleep disturbances and longer sleep latency appear to be tapping into poorer global cognition, whereas poorer subjective sleep quality and sleep efficiency are tapping into worse episodic memory and attention, respectively. These findings highlight the importance of assessing self‐reported sleep health in populations at higher risk for both sleep disorders and cognitive dysfunction.

